# Cardiovascular risk is similar in patients with glomerulonephritis compared to other types of chronic kidney disease: a matched cohort study

**DOI:** 10.1186/s12882-017-0511-z

**Published:** 2017-03-20

**Authors:** Holly L. Hutton, Adeera Levin, Jagbir Gill, Ognjenka Djurdjev, Mila Tang, Sean J. Barbour

**Affiliations:** 1 Centre for Inflammatory Diseases, Monash University, Clayton, VIC Australia; 2 Dept of Nephrology, Monash Health, 246 Clayton Rd, Clayton, VIC 3168 Australia; 3 Division of Nephrology, University of British Columbia, Vancouver, BC Canada; 4 BC Provincial Renal Agency, Vancouver, BC Canada; 5 Centre for Health Evaluation and Outcome Sciences, St Paul’s Hospital, Vancouver, BC Canada

**Keywords:** Cardiovascular disease, Glomerulonephritis, Chronic kidney disease

## Abstract

**Background:**

Patients with chronic kidney disease (CKD) due to glomerulonephritis (GN) are thought to be at high risk for cardiovascular disease (CVD). However, no study has examined whether GN directly contributes to CV risk beyond the effects conferred by pre-existing traditional risk factors and level of renal function.

**Methods:**

Matched cohort study using the previously described prospective CanPREDDICT study cohort. 2187 patients with CKD (eGFR 15–45 ml/min/m^2^) from 25 Canadian centres were divided into GN vs non-GN cause of CKD. Patients on immunotherapy for GN were not included in the study. Standardized measures of CV risk factors, biomarkers and CV outcomes were recorded over 3 years of follow-up, with the primary outcome measure being time to first all-cause CV event.

**Results:**

In the overall cohort, CV events occurred in 25 (8.7%) of the GN group and 338 (17.8%) of the non-GN group (HR 0.45, 95% CI 0.30–0.67, *p* < 0.01). In a Cox regression multivariable model that included age, sex, prior diabetes and CVD, baseline eGFR and onset of renal replacement therapy, the risk of CV events was similar in the GN and non-GN groups (HR 0.71, 95% CI 0.47–1.08, *p* = 0.11). GN and non-GN patients were matched by age and using a propensity score including sex, prior diabetes and CVD and baseline eGFR. In the matched group, the risk of CV events was similar in GN vs non-GN patients (*N* = 25/271 (9.2%) in both groups, HR 1.01, 95% CI 0.05–1.77, *p* = 0.9). An interaction analysis showed that CRP, ACR and troponin conferred differing amounts of CV risk in the GN and non-GN groups.

**Conclusions:**

Patients with advanced CKD due to GN have a high 8.7% absolute 3-year risk of CVD, attributable to prior CV risk factors and level of kidney function rather than the GN disease itself.

**Electronic supplementary material:**

The online version of this article (doi:10.1186/s12882-017-0511-z) contains supplementary material, which is available to authorized users.

## Background

Patients with glomerulonephritis (GN) have been traditionally characterised as being at high risk of cardiovascular disease (CVD), and this has been recently reiterated in the 2012 KDIGO GN guidelines [1]. GN patients are recognised to develop traditional epidemiologic risk factors for CVD, including hypertension and hyperlipidemia [2–4], as well as novel CV risk factors such inflammation, endothelial dysfunction and proteinuria [2, 5–7]. In addition, GN patients frequently develop chronic kidney disease (CKD) with impaired kidney function. Irrespective of cause, CKD is associated with a high risk of CVD and CV death [8–10]. Prior studies investigating CVD in GN have not accounted for these risk factors [2, 11, 12], and as such it remains unknown whether the high risk of CVD in GN patients is attributable to the disease itself or to the presence of concurrent CVD risk factors and CKD with low renal function.

CanPREDDICT is a prospective Canadian cohort study of 2544 patients with GN and non-GN CKD with standardized assessment of CV risk factors, CVD outcomes and biomarkers of inflammation, endothelial dysfunction and proteinuria. We used the CanPREDDICT cohort to examine our primary hypothesis that the risk of CVD over 3 years in patients with GN is higher compared to those with non-GN causes of CKD, after accounting for traditional CVD risk factors and renal function. Because novel CV risk factors are proposed to be uniquely important in glomerular diseases [7, 13, 14], we additionally explored whether the association between CVD risk and proteinuria or biomarkers of inflammation and endothelial dysfunction is different in patients with GN compared to non-GN CKD.

## Methods

Details of the CanPREDDICT study have been previously described [15]. In brief, 2544 patients with CKD (eGFR 15–45 ml/min/m^2^) from 25 Canadian centres were recruited from 2008 to 2009, and were prospectively followed for 3 years for CV outcomes with standardized measurements of multiple biomarkers. Patients with GN on immunosuppression were specifically excluded from CanPREDDICT. We included in our analysis those patients from the CanPREDDICT study with no missing demographic or biomarker data at baseline and with a known cause of primary kidney disease.

### Data collection

All patients were categorized into 2 groups (GN and non-GN) based on their primary renal diagnosis provided at the time of recruitment into the CanPREDDICT cohort. The cause of primary kidney disease was chosen from a list of options by the nephrologist at the time of recruitment into the study, with qualifying descriptions provided in free text format. An investigator (HH) blinded to other data and outcome status reviewed the renal diagnosis details to classify patients into non-GN or GN groups. GNs were further categorized as IgA nephropathy, membranous nephropathy, lupus nephritis, anti-neutrophil cytoplasmic antibody (ANCA) associated  vasculitis, focal segmental glomerulosclerosis (FSGS) or GN unspecified. Demographics, medications, blood pressure (BP) and comorbidities were collected at baseline and every 6 months over the 3-year study period. Blood and urine samples were collected at baseline. Proteinuria was assessed using urine albumin to creatinine ratio (uACR). Biomarkers known to be associated with CVD in the general CKD population were measured as previously described, including high sensitivity C-reactive protein (CRP) [16], troponin I [17], asymmetric dimethylarginine (ADMA) [18], interleukin-6 (IL-6) [19] and N-terminal pro-brain natriuretic peptide (ProBNP) [15–17].

### Definitions of outcomes

The primary outcome was the first occurrence of an all-cause CV event. All CV outcomes were centrally adjudicated based on source documentation by a blinded panel of three investigators using standardized definitions as previously described [15]. An all-cause CV event was defined as fatal or non-fatal myocardial infarction (MI), need for coronary revascularization (coronary artery bypass graft/percutaneous coronary intervention/percutaneous transluminal coronary angioplasty), ischemic stroke or congestive heart failure.

### Statistical analysis

Analysis of the primary outcome was based on the time from entry in the CanPREDDICT study to the first occurrence of an all-cause CV event, censored at death or the end of follow-up.

#### Comparing the risk of CV events in GN compared to non-GN CKD patients in the non-matched cohort

Survival without the primary CV endpoint was described in GN vs non-GN patients using the Kaplan Meier method and compared using the long-rank test. To control for renal function and traditional CV risk factors, we used Cox regression multivariable models that included GN vs non-GN CKD, age, sex, prior diabetes and CVD, baseline eGFR and onset of RRT as a time-dependent variable (to account for any confounding effect of differential progression to end stage renal disease on the risk of CV events).

#### Comparing the risk of CV events in GN to a matched cohort of non-GN CKD patients

We matched GN to non-GN patients using a stepwise approach: first direct matching on age (+/− 2.5 years), and second using a propensity score that included gender, history of diabetes, CVD and baseline eGFR. Matching was 1:1 using a calliper width of 0.25 standard deviation and a best-overall fit algorithm. Using the matched cohort, time to first all-cause CV event censored at death or end of follow-up was compared in GN vs non-GN CKD patients using a shared frailty Cox model to account for clustering on matched pairs.

#### Comparing the association of select biomarkers and CV risk in GN and non-GN patients

To investigate differences in the association between select biomarkers and CV risk in the GN and non-GN groups, an interaction term between each biomarker and disease type was included with the main effects in the matched cohort models. Using these models, we expressed the hazard ratios for the association between each biomarker and CVD separately in the GN and non-GN groups.

Because of the significant impact of diabetes on CVD risk, we performed stratified analyses in which the matching algorithm and analysis were repeated in subgroups with and without diabetes to explore consistency of results. The existing literature suggests that hypertension and dyslipidemia may be both directly caused by GN and result in CVD [4, 11, 14, 20–22]. As such, these two variables can be considered in the causal pathway between GN and CVD, and so we did not include them in our primary analyses. However, we performed a sensitivity analysis that included adjustment for both blood pressure and cholesterol levels. Variables with highly skewed distributions were transformed to the natural logarithm scale. Categorical variables were described as frequency (count) and compared across groups using Fischer’s exact test. Continuous variables with normal distributions were described as mean [standard deviation] and compared across groups using the t-test, and variables with non-normal distributions were described as median [interquartile range, IQR] and compared using the Wilcoxon Rank Sum test. All analyses were performed using SAS software, version 9.3 (SAS Institute Inc., Cary, NC, USA) and R software, version 3.1.0. All tests were two-sided with *P*-values <0.05 considered statistically significant.

## Results

**Fig. 1:**
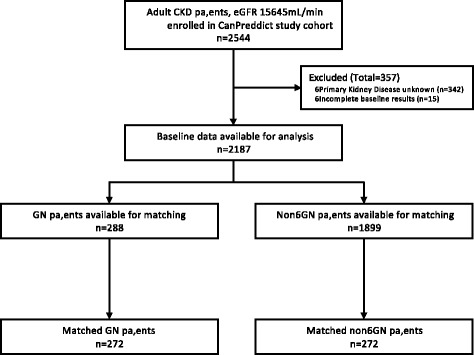
Flow diagram of patients included in the analytic cohort

There were 2544 patients in the CanPREDDICT study with 2187 in the analytic cohort, including 288 with GN and 1899 with non-GN CKD (see Fig. 1). Baseline characteristics of the cohort in the GN compared to non-GN patients are shown in Table 1. GN patients had less traditional CV risk factors, with younger age (58.9 vs 69.7 years, *p* < 0.01), less frequent diabetes (20% vs 54%, *p* < 0.001) and prior history of CVD (28% vs 47%, *p* < 0.01), and lower mean systolic BP (131 vs 134 mmHg, *p* < 0.01). GN patients had significantly higher uACR (59.5 vs 14.4 mmol/L, *p* < 0.01) and total cholesterol (179 vs 161 mg/dL, *p* = 0.03); lower ProBNP (273 vs 511 pg/mL, *p* < 0.01), IL-6 (3.04 vs 4.54, *p* < 0.01) and CRP (2.2 vs 3.0, *p* < 0.01) but similar eGFR (27.1 vs 27.7 ml/min/1.73 m^2^). Progression to RRT during follow-up was more common in the GN compared to non-GN group (25.4% vs 15.8%, *p* < 0.01).

**Table 1:** Characteristics of the GN and non-GN patients in the cohort. Data are presented as mean ± standard deviation (SD), median (IQR) or count (frequency)

Variable	GN	Non-GN	*P*-value
Number	288	1899	
Median follow up (months)	39.0 (33.5–39.0)	39.0 (22.5–39.0)	
Age (years)	58.9 ± 15.1	69.7 ± 11.7	<0.001
Male (%)	189 (66%)	1184 (62%)	0.3
Caucasian (%)	241 (84%)	1703 (90%)	<0.001
Primary cause of kidney disease (%)			
*Diabetes*	-	777 (41%)	
*Hypertension*	-	823 (48%)	
*PCKD*	-	108 (6%)	
*Other non-GN*	-	252 (13%)	
*GN subtypes (%)*			
*IgA Nephropathy*	61 (21%)	-	
*FSGS*	35 (12%)	-	
*ANCA Vasculitis*	21 (7%)	-	
*Lupus Nephritis*	13 (5%)	-	
*Membranous Nephritis*	9 (3%)	-	
*GN Unspecified*	149 (52%)	-	
Diabetes (%)	58 (20%)	1025 (54%)	<0.001
CVD history (%)			<0.001
*No CVD*	206 (72%)	998 (53%)	
*Ischemic HD*	33 (12%)	365 (17%)	
*CHF*	29 (10%)	215 (11%)	
*Ischemic and CHF*	20 (7%)	321 (17%)	
Mean eGFR (ml/min/1.73 m^2^) ++/SD	27.1 ± 9.1	27.7 ± 8.9	0.3
eGFR categories (%)			0.1
*< 20 ml/min*	81 (28%)	428 (23%)	
*20–29*	107 (37%)	757 (40%)	
*> 30*	100 (35%)	714 (40%)	
uACR (mg/mmol)	59.5 [13.7–172.3]	14.4 [2.9–76.8]	<0.001
Systolic BP (mmHg)	131 ± 18	134 ± 20	<0.001
Diastolic BP (mmHg)	75 ± 12	70 ± 12	<0.001
Albumin (g/L)	40.0 ± 4.8	40.4 ± 4.2	0.08
Total cholesterol (mg/dL)	4.6 ± 1.2	4.2 + 1.1	0.03
Elevated Troponin I (% > LLD)	71 (25%)	714 (38%)	<0.001
CRP (mg/mL)	2.2 [0.9–5.3]	3.0 [1.2–6.9]	<0.001
ADMA	0.54 ± 0.94	0.55 ± 0.12	0.1
IL-6 (μg/L)	3.04 [1.00–5.80]	4.54 [1.00–7.25]	<0.001
NT-ProBNP (pg/mL) Pg/mL	273 [119–727]	511 [213–1485]	<0.001

Abbreviations: *PCKD* polycystic kidney disease, *FSGS* focal segmental glomerulosclerosis, *CVD* cardiovascular disease, *CHF* congestive heart failure, *BP* blood pressure, *LLD* lower limit of detection, *ADMA* asymmetric dimethylarginine, *IL-6* interleukin 6, *NT-Pro-BNP* N-terminal pro-brain natriuretic peptide

### The risk of CVD in GN vs non-GN CKD patients in the non-matched cohort

**Fig. 2:**
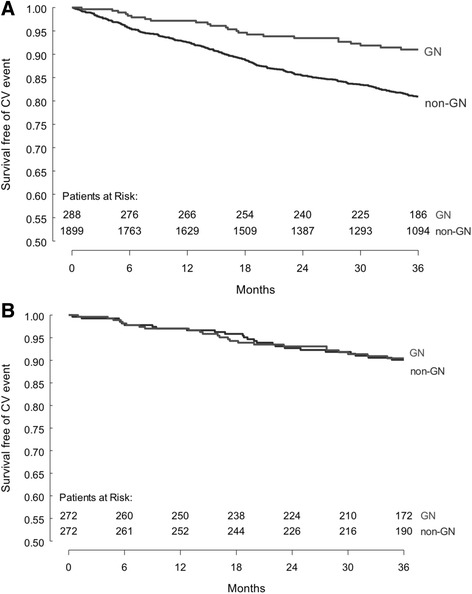
The probability of survival without an all-cause CV event in GN compared to non-GN patients in **a**) the overall cohort prior to matching (log-rank *p*-values <0.01), and **b**) in the matched cohort (log-rank *p*-values = 0.96)

There were a total of 363 CV events in the entire cohort over the 3-year period, with 25 events in the GN group (8.7%), and 338 events (17.8%) in the non-GN group. The most common event was fatal or non-fatal MI (*N* = 166, including 9 GN and 157 non-GN), followed by CHF (*N* = 122, including 8 GN and 114 non-GN), stroke (*N* = 51, including 5 GN and 46 non-GN) and coronary revascularization (*N* = 24, including 3 GN and 21 non-GN). Figure 2a outlines the CV event free survival in GN compared to non-GN patients. GN patients had superior CV event free survival compared to non-GN patients (unadjusted HR = 0.45, 95% CI 0.3–0.67, *p* < 0.01). Table 2 displays the result of the multivariate model for the risk of CV event in GN versus non-GN patients. After adjustment for potential measured confounders, the risk of CV events was similar in the two groups (HR = 0.71, 95% CI 0.47–1.08, *p* = 0.11). In a sensitivity analysis, when mean arterial blood pressure and total cholesterol were added to the model the results were unchanged (data not shown).

**Table 2:** The results of univariable and multivariable Cox regression models for the risk of all-cause CV events in the overall non-matched cohort

	HR	95%CI	*P*-value
Univariable Model			
GN vs non-GN CKD	0.45	0.30–0.67	<0.001
Multivariable Model			
GN vs non-GN CKD	0.71	0.47–1.08	0.1
Age	1.03	1.02–1.04	<0.001
Male sex	1.05	0.85–1.31	0.6
Diabetes	1.71	1.37–2.13	<0.001
Prior CVD	2.28	1.82–2.87	<0.001
Baseline eGFR	0.97	0.96–0.99	<0.001
RRT^a^	2.01	1.38–2.93	<0.001

^a^ as a time-dependent variable

### The risk of CVD in GN compared to a matched cohort of non-GN CKD patients

To further control for renal function and CV risk factors in the association of GN and CV risk, we matched 272 patients from the GN group with 272 patients from the non-GN group based on age, sex, eGFR and prior diabetes and CVD. Characteristics of the matched cohort are shown in Table 3. Differences in age, BP, prior diabetes and CVD and total cholesterol between GN and non-GN patients that were seen in the overall cohort were no longer present after matching. GN patients had higher uACR (58.7 vs 17.2 mg/mmol, *p* < 0.01) and lower serum albumin (40 vs 41 g/L *p* < 0.01), but there were no significant differences in troponin I, IL-6, Pro-BNP, ADMA or CRP between the groups (Table 3). Progression to RRT during follow-up occurred in 24.6% of the non-GN group and 25.4% of the GN group (*p* = 0.84) in the matched cohort.

**Table 3:** Characteristics of matched GN and non-GN groups. Results are presented as mean ± standard deviation, median (IQR) or count (frequency)

Variable	GN	Non-GN	*P* value
Number	272	272	
Median follow up	39.0 (33.2–39.0)	39.0 (33.8–39.0)	
Age (years)	60.5 ± 14	60.4 ± 14	0.9
Male	177 (65%)	177 (65%)	0.9
Caucasian	231 (85%)	242 (89%)	0.4
Cause of Kidney Disease			
*Diabetic*	-	49 (18%)	
*Hypertensive*	-	87 (32%)	
*PCKD*	-	44 (16%)	
*Other non-GN*	-	92 (34%)	
*IgA*	60 (22%)	-	
*FSGS*	33 (12%)	-	
*ANCA Vasculitis*	21 (8%)	-	
*Lupus*	13 (5%)	-	
*Membranous Nephritis*	9 (3%)		
*GN unspecified*	136 (50%)		
Diabetes	57 (21%)	60 (22%)	0.7
CVD history			0.9
*No CVD*	193 (71%)	201 (74%)	
*Ischemic*	33 (12%)	30 (11%)	
*CHF*	27 (10%)	25 (9%)	
*Ischemic and CHF*	19 (7%)	16 (6%)	
eGFR (ml/min/1.73 m^2^)	27.0 ± 9.1	27.2 ± 9.1	0.8
eGFR categories			0.7
*< 20*	76 (28%)	68 (25%)	
*20–29*	103 (38%)	109 (40%)	
*> 30*	93 (34%)	95 (35%)	
ACR (mg/mmol)	58.7 [13.6, 170.2]	17.2 [3.6–90.0]	<0.001
Systolic BP (mmHg)	132 ± 19	131 ± 19	0.8
Diastolic BP (mmHg)	75 ± 12	74 ± 12	0.4
Statin	158 (58%)	147 (54%)	0.3
Albumin (g/L)	39.8 ± 4.8	41.1 ± 4.2	<0.001
Total cholesterol (mg/dL)	4.7 ± 1.2	4.5 ± 1.4	0.4
Troponin I (> LLD)	71 (26%)	68 (25%)	0.8
CRP (mg/mL)	2.4 [0.9–5.6]	2.4 [1.1–5.5]	0.6
ADMA	0.537 ± 0.094	0.534 ± 0.151	0.9
IL-6 (μg/L)	3.55 [1.00–6.02]	3.5 [1.00–5.27]	0.6
NT-ProBNP (pg/mL) Pg/mL	299 [121–799]	291 [107–757]	0.7

Abbreviations: *PCKD* polycystic kidney disease, *FSGS* focal segmental glomerulosclerosis, *CVD* cardiovascular disease, *CHF* congestive heart failure, *BP* blood pressure, *LLD* lower limit of detection, *ADMA* asymmetric dimethylarginine, *IL-6* interleukin 6, *NT-Pro-BNP* N-terminal pro-brain natriuretic peptide

The frequency of CV events over 3 years in the matched cohort was 9.2% (*N* = 25) in both the GN and non-GN groups. Figure 2b shows that the CV event free survival was similar in the GN patients compared to the matched cohort of non-GN patients (log-rank *p*-value 0.96). The risk of CV events was similar in the GN compared to non-GN patients in both univariable (HR 1.01, 95% CI 0.58–1.77, *p* = 0.96) and multivariable models (HR 0.99, 95% CI 0.56–1.75, *p* = 0.96, see Table 4). Because of residual differences in proteinuria between the groups after matching, we additionally added uACR to the multivariable model which did not change our results (GN vs non-GN HR 0.87, 95% CI 0.49–1.58, *p* = 0.66). In a sensitivity analysis, when mean arterial blood pressure and total cholesterol were added to the model the results were unchanged (data not shown).

**Table 4:** The results of univariable and multivariable shared frailty Cox regression models for the risk of all-cause CV events in the matched cohort

	HR	95%CI	*P*-value
Univariable Model			
GN vs non-GN CKD	1.01	0.58–1.77	0.9
Multivariable Model			
GN vs non-GN CKD	0.99	0.56–1.75	0.9
Age	1.03	1.01–1.07	0.03
Male sex	1.27	0.63–2.55	0.5
Diabetes	3.11	1.59–6.07	<0.001
Prior CVD	3.16	1.55–6.46	<0.001
Baseline eGFR	1.01	0.98–1.05	0.4
RRT ^a^	1.51	0.51–4.50	0.4

^a^ as a time-dependent variable

We repeated the matching and analysis in diabetic and non-diabetic subgroups. Of the 112 matched patients with diabetes, 21.4% (*n* = 12) of both the GN and non-GN patients had a CV event. Of the 388 matched patients without diabetes, CV events occurred in 6.2% (*N* = 12) and 8.2% (*N* = 16) in the GN and non-GN groups respectively. In multivariable models, there was no difference in the risk of CV events between GN and non-GN patients in either the diabetic or non-diabetic subgroups (data not shown).

### Biomarkers as CV risk factors in GN compared to matched non-GN CKD patients

Using the matched cohort, we explored whether the risk of CVD associated with ACR, CRP, IL-6, ADMA, troponin I and ProBNP was different in the GN compared to non-GN groups using interaction terms. The hazard ratios for each biomarker by GN group are presented in Table 5 and main effects in Additional file 1: Table S1. Although the interaction terms were not statistically significant (*p*-values 0.06–0.94), there was a suggestion of quantitative differences in the hazard ratios for uACR, CRP and troponin I in the GN compared to non-GN groups (interaction *p*-values 0.15, 0.24 and 0.06 respectively). The hazard ratio for the association between uACR and CV risk was higher and statistically significant in the non-GN group but not in the GN group. In comparison the opposite was true for CRP, which was associated with CV risk only in GN patients. Troponin I was a strong risk factor for CVD in both groups, but the magnitude of risk was three times greater in non-GN compared to GN patients.

**Table 5:** In the matched cohort, the association between each biomarker and the risk of all-cause CV events in the GN and non-GN groups using multivariable models that included interaction terms between the GN vs non-GN group and each biomarker

Biomarker		HR	95% CI
uACR (per log unit)	GN	1.02	0.81–1.30
	Non-GN	1.30	1.03–1.62
ADMA (per 1StD)	GN	1.54	0.95–2.50
	Non-GN	1.06	0.82–1.37
ProBNP (per 1StD)	GN	2.21	1.43–3.40
	Non-GN	2.54	1.68–3.86
CRP (per 1StD)	GN	1.70	1.11–2.60
	Non-GN	1.22	0.81–1.84
IL6 (per 1StD)	GN	1.57	1.12–2.22
	Non-GN	1.43	0.95–2.16
Troponin I (>LLD vs. <LLD)	GN	3.57	1.55–8.22
	Non-GN	12.16	4.64–31.87

uACR, ProBNP, CRP and IL6 were log-transformed for analysis

Abbreviations: *LLD* lower limit of detection, *StD* standard deviation

## Discussion

Although guidelines state that GN patients should be considered high risk for CVD [1], this is based on conflicting studies that did not account for renal function, and therefore the contribution of CKD and pre-existing traditional risk factors to CVD risk in glomerular diseases remained unknown. In order to address this deficiency, we used a large prospective CKD cohort with standardized measures of CV risk factors and outcome events to investigate the risk of CVD in GN compared to non-GN patients matched for renal function and prior CV risk factors. We show that GN patients are at a high 9.2% absolute risk of CVD over 3 years, but that in contrast to our a priori hypothesis this risk was not different from otherwise comparable patients with advanced CKD without GN. These results suggest that once a sustained reduction in kidney function has developed, GN is not independently associated with additional CV risk beyond that explained by reduced eGFR and pre-existing traditional risk factors.

The assumption that primary GN is linked to an increased risk of vascular events derives from older case series with conflicting results, ranging from no increased risk to an 85 fold relative risk of CVD compared to the general population [20, 23–27]. More recent cohort studies described a more attenuated 2–8 fold relative risk in CVD, but none accounted for traditional CV risk factors or the degree of renal dysfunction [2, 4, 11, 12, 28]. The association of eGFR with CVD has been well established in both the general CKD and GN populations [2, 4, 5, 9, 10]. This is the first study to systematically compare GN to non-GN patients while considering both baseline eGFR and prior traditional CV risk factors. In unadjusted analysis, the GN patients had a significantly lower risk of CV events compared to the non GN patients, likely due to their younger age and comparative lack of traditional CV risk factors. However, after accounting for age, sex, diabetes, prior CVD and eGFR using two different methods (multivariable adjusted models and a two-stage propensity score matching algorithm), we showed that CKD patients with GN had a similar risk of CVD compared to non-GN patients. Because our results were unchanged when we adjusted for RRT, our findings are not likely confounded by differential rates of progression to ESRD. In our primary analyses we did not adjust for blood pressure and dyslipidemia because these may both mediate the risk of CVD that results from GN. However, in sensitivity analyses that included both of these variables our results were unchanged. Nearly 50% of patients with non-GN CKD had diabetic nephropathy, implying severe diabetes that may disproportionately contribute to CVD risk in a way not fully accounted for by the propensity score. To address this, we performed sensitivity analyses in which we repeated the matching algorithm in subgroups based on diabetic status. As expected, we observed substantially higher CVD risk in diabetics compared to non-diabetics (21.4% vs. 6.2–8.2% respectively), but in both subgroups CV event rates were similar in GN and non-GN patients. Using a systematic and comprehensive analysis strategy, we have shown that CKD patients with GN are at a high absolute 3-year risk of CVD, but that this is explained by the severity of renal dysfunction and the accumulation of traditional CV risk factors rather than being attributed to the GN disease itself.

Because CVD is such an important cause of mortality in patients with CKD [8, 9, 14, 21, 29], our findings have substantial implications to the clinical care and management of CVD in GN patients. Cardiac risk stratification is especially important in younger patients, being necessary to implement appropriate prevention strategies, and in the assessment for kidney transplantation. Our unmatched GN group had a high 8.2% absolute 3-year risk of CVD despite a mean age of only 58 years, and 72–80% having no prior history of DM or CVD. Prevention strategies such as statin therapy are not likely to be employed as routine treatment for these patients, owing to their lack of comorbidities and relatively young age. However, our study shows that GN patients with CKD are at high absolute CV risk, greater than 10% over 10 years, and therefore should be considered for statin therapy according to the KDIGO lipid guidelines [30].

Although biomarkers of inflammation and vascular health have been associated with CVD in the general CKD population, our study offers novel insights into the possibility that there are differences in the predictive value of biomarkers in those with GN compared to non-GN CKD. Our interaction analyses suggested a non-significant trend towards CRP being a stronger CVD risk factor in CKD patients with GN, and uACR and troponin being more important CVD risk factors in non-GN CKD. Reasons for this are not clear, but may include inflammation and CRP being more pronounced in GN thereby playing a more prominent role in the development of CVD; albuminuria reflecting local renal pathology in GN patients instead of being a marker of endothelial dysfunction and CV risk as it is in the general and all-cause CKD populations; and [5, 6, 10, 31]. more common non-CVD causes of increased troponin in patients with GN [32–34]. These results require confirmation in larger studies with sufficient power to investigate and explain differential associations between biomarkers and CVD risk within subgroups of CKD etiology.

Our GN group comprised of patients with advanced kidney disease with a mean eGFR of 27 ml/min/1.73 m^2^ and who were not on immunotherapy, which must be considered in the generalizability of our results. Compared to other cohorts of patients with active GN such as the Toronto GN registry [35], our patients are older, with lower eGFR and lower levels of proteinuria, consistent with study enrolment at a later stage in the disease process. Low levels of CRP (2.4 μg/L) and IL-6 (3.55 μg/L) were seen in the GN patients and were comparable to levels in the matched non-GN group. Our study clearly shows that when glomerular disease becomes quiescent, GN patients are at comparable CV risk to other patients with CKD and similar CV risk factors. Future research is required to determine if our results apply at an early stage of glomerular disease, in which renal function is preserved but more severe inflammation or proteinuria have been hypothesized to disproportionately contribute to CVD in the absence of other CV risk factors. Although no study has specifically addressed this issue, the results of Mahmoodi et al. suggest this may not be the case. In this study of 298 nephrotic patients with mean eGFR of 59 ml/min/1.73 m^2^, the absolute risk of CVD was high at 1.48% per year, however this was disproportionately dominated by those with diabetic nephropathy. In the subgroup of GN patients without diabetes or prior CVD, the risk was only 0.82% per year, suggesting that even amongst nephrotic patients with preserved renal function, the development of CVD is substantially related to pre-existing CV risk factors [2]. The considerably higher incidence of CV events seen in our GN cohort compared to that of Mahmoodi, at 3.12% per year, is probably a result of older age, lower eGFR and greater accumulation of traditional CV risk factors. If future research confirms our finding that CVD in patients with early GN is predominantly determined by prior traditional risk factors and level of renal function, then GN patients without other comorbidities and normal renal function may not have an increased absolute risk of CVD. This finding would substantially impact therapeutic decisions regarding primary prevention strategies with statins at earlier stages of glomerular disease.

Our study has several other limitations that should be considered in the interpretation of the results. The specific type of glomerular disease was unknown in approximately 50% of the GN patients. This limits our ability to draw conclusions about specific GN types and CV risk. Certain types of GN such as minimal change disease may not be associated with CVD due to infrequent nephrotic flares with long periods of intermittent disease quiescence [2, 27]. However, it is unlikely that such patients would have been included in our GN cohort since progression to advanced CKD is unlikely in the absence of persistent ongoing proteinuria and disease activity. There was no available information about cigarette smoking as a CV risk factor, and so this could not be included in our multivariable models. Centrally adjudicated peripheral vascular disease outcome events were not available for analysis; however, our composite CV outcome event definition is nonetheless consistent with that used in major CV clinical trials [36–38]. Finally, it is possible that the GN and non-GN groups will differ in CV events occurring late after study enrolment, and this difference would not be detected by the 3 year follow up of our study.

## Conclusions

We have shown that GN patients with CKD and decreased eGFR who are not on active immunosuppressive therapy have a high 9.2% 3 year risk of CV events. However, this risk does not appear to be different from otherwise similar CKD patients without GN, suggesting that the elevated risk of CVD in GN patients may be attributable to prior CV risk factors and level of kidney function rather than the GN disease itself.

## Additional file

Additional file 1: Table S1. The biomarker hazard ratios using Cox proportional hazards models that included GN vs non-GN CKD and each biomarker individually. ACR, CRP, IL-6, ProBNP and FGF-23 were log-transformed for analysis. (DOCX 15 kb)

## Abbreviations

ADMA: Asymmetric dimethylarginine
BP: Blood pressure
CKD: Chronic kidney disease
CRP: C-reactive protein
CVD: Cardiovascular disease
FSGS: Focal segmental glomerulosclerosis
GN: Glomerulonephritis
IL-6: Interleukin-6
MI: Myocardial infarction
Pro-BNP: N-terminal pro-brain natriuretic peptide
RRT: Renal replacement therapy
uACR: Urine albumin to creatinine ratio

## References

[1] Kidney Disease: Improving Global Outcomes (KDIGO) Glomerulonephritis Work Group (2012). KDIGO clinical practice guideline for glomerulonephritis. Kidney Int.

[2] Mahmoodi BK, ten Kate MK, Waanders F, Veeger NJ, Brouwer JL, Vogt L, Navis G, van der Meer J (2008). High absolute risks and predictors of venous and arterial thromboembolic events in patients with nephrotic syndrome: results from a large retrospective cohort study. Circulation.

[3] Ihm CG (2015). Hypertension in chronic glomerulonephritis. Electrolytes Blood Press.

[4] Myllymaki J, Syrjanen J, Helin H, Pasternack A, Kattainen A, Mustonen J (2006). Vascular diseases and their risk factors in IgA nephropathy. Nephrol Dial Transplant.

[5] Matsushita K, van der Velde M, Astor BC, Woodward M, Levey AS, de Jong PE, Coresh J, Gansevoort RT (2010). Association of estimated glomerular filtration rate and albuminuria with all-cause and cardiovascular mortality in general population cohorts: a collaborative meta-analysis. Lancet.

[6] Astor BC, Matsushita K, Gansevoort RT, van der Velde M, Woodward M, Levey AS, Jong PE, Coresh J, Astor BC, Matsushita K (2011). Lower estimated glomerular filtration rate and higher albuminuria are associated with mortality and end-stage renal disease. A collaborative meta-analysis of kidney disease population cohorts. Kidney Int.

[7] Matsuzawa Y, Guddeti RR, Kwon TG, Lerman LO, Lerman A (2014). Treating coronary disease and the impact of endothelial dysfunction. Prog Cardiovasc Dis.

[8] Bello AK, Hemmelgarn B, Lloyd A, James MT, Manns BJ, Klarenbach S, Tonelli M (2011). Associations among estimated glomerular filtration rate, proteinuria, and adverse cardiovascular outcomes. Clin J Am Soc Nephrol.

[9] Go AS, Chertow GM, Fan D, McCulloch CE, Hsu CY (2004). Chronic kidney disease and the risks of death, cardiovascular events, and hospitalization. N Engl J Med.

[10] van der Velde M, Matsushita K, Coresh J, Astor BC, Woodward M, Levey A, de Jong P, Gansevoort RT, van der Velde M, Matsushita K (2011). Lower estimated glomerular filtration rate and higher albuminuria are associated with all-cause and cardiovascular mortality. A collaborative meta-analysis of high-risk population cohorts. Kidney Int.

[11] Heaf J, Lokkegaard H, Larsen S (1999). The epidemiology and prognosis of glomerulonephritis in Denmark 1985–1997. Nephrol Dial Transplant.

[12] Orodonez JHR, Kilebrew E, Fireman B (1993). The increased risk of coronary heart disease associated with nephrotic syndrome. Kidney Int.

[13] Hansson GK (2005). Inflammation, atherosclerosis, and coronary artery disease. N Engl J Med.

[14] Liu M, Li XC, Lu L, Cao Y, Sun RR, Chen S, Zhang PY (2014). Cardiovascular disease and its relationship with chronic kidney disease. Eur Rev Med Pharmacol Sci.

[15] Levin A, Rigatto C, Brendan B, Madore F, Muirhead N, Holmes D, Clase CM, Tang M, Djurdjev O, Can P (2013). Cohort profile: Canadian study of prediction of death, dialysis and interim cardiovascular events (CanPREDDICT). BMC Nephrol.

[16] Vickery S, Webb MC, Price CP, John RI, Abbas NA, Lamb EJ (2008). Prognostic value of cardiac biomarkers for death in a non-dialysis chronic kidney disease population. Nephrol Dial Transplant.

[17] Hickman PE, McGill DA, Talaulikar G, Hiremagalur B, Bromley J, Rahman A, Koerbin G, Southcott E, Potter JM (2009). Prognostic efficacy of cardiac biomarkers for mortality in dialysis patients. Intern Med J.

[18] Young JM, Terrin N, Wang X, Greene T, Beck GJ, Kusek JW, Collins AJ, Sarnak MJ, Menon V (2009). Asymmetric dimethylarginine and mortality in stages 3 to 4 chronic kidney disease. Clin J Am Soc Nephrol.

[19] Barreto DV, Barreto FC, Liabeuf S, Temmar M, Lemke HD, Tribouilloy C, Choukroun G, Vanholder R, Massy ZA (2010). European Uremic Toxin Work G: Plasma interleukin-6 is independently associated with mortality in both hemodialysis and pre-dialysis patients with chronic kidney disease. Kidney Int.

[20] Vosnides G, Cameron JS (1974). Hyperlipidemia in renal disease. Med J Aust.

[21] Alani H, Tamimi A, Tamimi N (2014). Cardiovascular co-morbidity in chronic kidney disease: Current knowledge and future research needs. World J Nephrol.

[22] Wanner C (2000). Importance of hyperlipidaemia and therapy in renal patients. Nephrol Dial Transplant.

[23] Alexander JH, Schapel GJ, Edwards KD (1974). Increased incidence of coronary heart disease associated with combined elevation of serum triglyceride and cholesterol concentrations in the nephrotic syndrome in man. Med J Aust.

[24] Gilboa N (1976). Incidence of coronary heart disease associated with nephrotic syndrome. Med J Aust.

[25] Berlyne GM, Mallick NP (1969). Ischaemic heart-disease as a complication of nephrotic syndrome. Lancet.

[26] Wass VJ, Jarrett RJ, Chilvers C, Cameron JS (1979). Does the nephrotic syndrome increase the risk of cardiovascular disease?. Lancet.

[27] Hopper J, Ryan P, Lee JC, Rosenau W (1970). Lipoid nephrosis in 31 adult patients: renal biopsy study by light, electron, and fluorescence microscopy with experience in treatment. Medicine.

[28] Faurschou M, Mellemkjaer L, Starklint H, Kamper AL, Tarp U, Voss A, Jacobsen S (2011). High risk of ischemic heart disease in patients with lupus nephritis. J Rheumatol.

[29] Kidney Disease: Improving Global Outcomes (KDIGO) CKD Work Group (2013). KDIGO clinical practice guideline for the evaluation and management of chronic kidney disease. Kidney Int.

[30] Kidney Disease: Improving Global Outcomes (KDIGO) Lipid Work Group (2013). KDIGO clinical practice guideline for lipid management in chronic kidney disease. Kidney Int.

[31] Matsuzawa Y, Lerman A (2014). Endothelial dysfunction and coronary artery disease: assessment, prognosis, and treatment. Coron Artery Dis.

[32] Hamwi SM, Sharma AK, Weissman NJ, Goldstein SA, Apple S, Canos DA, Pinnow EE, Lindsay J (2003). Troponin-I elevation in patients with increased left ventricular mass. Am J Cardiol.

[33] Paradis P, Dali-Youcef N, Paradis FW, Thibault G, Nemer M (2000). Overexpression of angiotensin II type I receptor in cardiomyocytes induces cardiac hypertrophy and remodeling. Proc Natl Acad Sci U S A.

[34] Urushihara M, Kinoshita Y, Kondo S, Kagami S (2012). Involvement of the intrarenal renin-angiotensin system in experimental models of glomerulonephritis. J Biomed Biotechnol.

[35] Barbour SJ, Greenwald A, Djurdjev O, Levin A, Hladunewich MA, Nachman PH, Hogan SL, Cattran DC, Reich HN (2012). Disease-specific risk of venous thromboembolic events is increased in idiopathic glomerulonephritis. Kidney Int.

[36] Group SR, Wright JT, Williamson JD, Whelton PK, Snyder JK, Sink KM, Rocco MV, Reboussin DM, Rahman M, Oparil S (2015). A randomized trial of intensive versus standard blood-pressure control. N Engl J Med.

[37] Estruch R, Ros E, Salas-Salvado J, Covas MI, Corella D, Aros F, Gomez-Gracia E, Ruiz-Gutierrez V, Fiol M, Lapetra J (2013). Primary prevention of cardiovascular disease with a Mediterranean diet. N Engl J Med.

[38] Investigators O, Yusuf S, Teo KK, Pogue J, Dyal L, Copland I, Schumacher H, Dagenais G, Sleight P, Anderson C (2008). Telmisartan, ramipril, or both in patients at high risk for vascular events. N Engl J Med.

