# Supplementary material for: Cardiovascular risk is similar in patients with glomerulonephritis compared to other types of chronic kidney disease: a matched cohort study
Source: BMC Nephrol. 2017 Mar 20;18:95. doi: 10.1186/s12882-017-0511-z (PMC5358048; doi:10.1186/s12882-017-0511-z)
Supplement: Additional file 1: Table S1. — The biomarker hazard ratios using Cox proportional hazards models that included GN vs non-GN CKD and each biomarker individually. ACR, CRP, IL-6, ProBNP and FGF-23 were log-transformed for analysis. (DOCX 15 kb) [file 12882_2017_511_MOESM1_ESM.docx]

**Table S1:** The biomarker hazard ratios using Cox proportional hazards models that included GN vs non-GN CKD and each biomarker individually. ACR, CRP, IL-6, ProBNP and FGF-23 were log-transformed for analysis.

|  | HR | 95%CI | P-value |
| --- | --- | --- | --- |
|  | | | |
| GN vs non-GN CKD | 0.89 | 0.50-1.59 | 0.7 |
| uACR (per log unit) | 1.16 | 0.98-1.37 | 0.07 |
|  | | | |
| GN vs non-GN CKD | 1.03 | 0.59-1.80 | 0.9 |
| ADMA (per 1StD) | 1.14 | 0.94-1.38 | 0.1 |
|  | | | |
| GN vs non-GN CKD | 1.01 | 0.57-1.78 | 0.9 |
| ProBNP (per 1StD) | 2.38 | 1.75-3.22 | < 0.001 |
|  | | | |
| GN vs non-GN CKD | 0.99 | 0.57-1.75 | 0.9 |
| CRP (per 1StD) | 1.44 | 1.07-1.94 | 0.01 |
|  | | | |
| GN vs non-GN CKD | 0.95 | 0.54-1.68 | 0.9 |
| IL6 (per 1StD) | 1.51 | 1.16-1.97 | <0.001 |
|  | | | |
| GN vs non-GN CKD | 0.99 | 0.56-1.76 | 0.9 |
| Troponin I (>LLL vs. <LLL) | 6.31 | 3.39-11.75 | <0.001 |

Abbreviations: ADMA, asymmetric dimethylarginine; ProBNP, N-terminal probrain natriuretic peptide; IL-6, interleukin 6; StD standard deviation; LLD, lower limit of detection
